# Selection of optimal therapeutic modality for early-stage extranodal natural killer/T-cell lymphoma patients under the guidance of single-nucleotide polymorphism signature

**DOI:** 10.17305/bjbms.2021.6419

**Published:** 2021-08-24

**Authors:** Dong-Hua Yang, Zhe-Sheng Chen

**Affiliations:** 1 Department of Pharmaceutical Sciences, St. John’s University, New York, USA; 2 Institute for Biotechnology, St. John’s University, New York, USA

The therapeutic modalities of early-stage and advanced extranodal natural killer/T-cell lymphoma (NKTCL) patients are completely different. The former is mainly radiotherapy with or without chemotherapy, while the latter relies on chemotherapy-based systemic treatment [1,2]. According to Ann Arbor staging system, approximately 70% of the NKTCL patients are classified as early-stage cases who are promising to be cured [3]. Considering NKTCL is sensitive to radiation but may be resistant to chemotherapy, the radiotherapy is considered to be the most important treatment for some early-stage patients with a satisfactory local control rate and could be used alone [4]. However, systemic recurrence after radiotherapy in a portion of NKTCL patients seriously affects their long-term survival, and the first-line treatment combined with radiotherapy and chemotherapy is considered necessary. Therefore, the use of radiotherapy alone in early-stage NKTCL is still a controversial issue [5,6].

Some studies have been devoted to exploring how to choose the optimal therapeutic modality for early-stage NKTCL patients, radiotherapy alone or combined radiotherapy with chemotherapy. A large-scale multicenter cohort study assessed the optimal combination and sequence of radiotherapy for NKTCL, Yang et al. found that risk-adapted therapy of radiotherapy alone is sufficient for low-risk early-stage NKTCL patients with no risk factors (age >60 years, ECOG > 2, stage II disease, elevated LDH, primary tumor invasion) [7]. Another study of Hong et al. established the first disease staging system specific to NKTCL, patients with stage I disease defined by the new system were recommended to receive radiotherapy alone [8]. The existing predictive models are all based on clinical indicators and have their limitations in revealing the nature of disease, and so there has been interest in the use of molecular markers to improve prognostication.

In the journal of Blood, Tian et al. did an international multicenter study to assess the predictive value of single-nucleotide polymorphism (SNP) signatures for progress-free survival (PFS) in patients with NKTCL [9]. The authors developed a seven-SNP-based classifier that can accurately calculate individualized prognostic risk scores. The prediction accuracy of this classifier was not influenced by tumor heterogeneity and tissue specificity, which can be used as a supplement to the current classical International Prognostic Index (IPI), the Korea Prognostic Index (KPI), and prognostic index of natural killer lymphoma (PINK) prognostic systems. A Nomogram based on the SNP classifier, combined with age, Ann Arbor stage and other clinical indicators, can further improve the prognosis prediction accuracy, and an online risk scoring calculation system is available. Moreover, the SNP classifier can also be used to guide clinical decision-making. For patients in Ann Arbor stage I, low-risk patients identified by the SNP classifier were recommended to receive radiotherapy alone, thereby reducing treatment-related side effects and saving costs, while the others in the early stage should receive combined radiotherapy and chemotherapy to avoid recurrence as much as possible. Therefore, this new tool provides clinical flexibility and enables individualized medical care, and it can also simplify clinical management decisions.

In the era of precision therapy, it is very important to incorporate molecular markers into clinical practice to promote individualized therapy so that patients will not be treated with a one-size-fits-all approach, avoiding overtreatment of some patients and under-treatment of others. Remarkably, Tian’s study is no longer limited to the use of clinicopathological prognostic indicators, but to conduct in-depth research from the gene level to explore new biomarkers, which provides a new insight for improving the accuracy of prognostic prediction in NKTCL and the guiding treatment strategy selection for early-stage NKTCL patients. After further elucidating the underlying mechanism and verifying the effectiveness of the model in prospective clinical trials, it is of great significance to apply it to clinical practice.
